# Exploring Consumers’ Interest in Choosing Sustainable Food

**DOI:** 10.3389/fpsyg.2020.00489

**Published:** 2020-04-22

**Authors:** Shih-Yun Hsu, Huai-Chen Wang, Juei-Ling Ho, Ho-Cheng Chen

**Affiliations:** ^1^Department of Business Management, National Taichung University of Science and Technology, Taichung, Taiwan; ^2^Department of Leisure Regimen Management, Tainan University of Technology, Tainan, Taiwan

**Keywords:** sustainable food, level of knowledge, family and friend support, price accessibility, health incentive, interest

## Abstract

In order for humans to achieve a healthier diet and maintain ecological balance, a new concept regarding sustainable food has been introduced. The aim of this study is, therefore, to explore consumers’ interest in choosing sustainable food. The study utilized “Do Survey,” an internet survey platform, to distribute questionnaires. Survey invitations were sent out in a snowball manner. Invitations were posted in multiple online communities and forwarded by various members to other sites; 333 valid responses were collected. The results show that family and friend support and health incentive are the two strongest predictors for sustainable food buying interest. Price accessibility, on the other hand, has no impact on interest. This is not to say that price accessibility does not influence behavior. It just means that it is not the factor that fosters interest. Once an individual is interested, she/he may still rely heavily on price accessibility in purchasing sustainable food.

## Introduction

The advancement of civilization has improved living standards, but advancement comes with certain undesired outcomes. For example, people nowadays tend to overindulge themselves; consequently, obesity is a common problem in many countries such as Taiwan (Hwang et al., 2006) and America (Maiano et al., 2016). Obesity can also lead to other forms of sickness and increase health risk (Manna and Jain, 2015; Danziger et al., 2016). According to a study (Oyebode et al., 2014) in the *British Medical Journal*, daily consumption of fruits and vegetables reduces the risk of health-related death by 42%, cancer by 25%, and heart problems by 31%. Despite the compelling findings, people are still prone to an unhealthy diet. It is therefore important to study their decision-making.

In the study of decision-making and behavioral intention, many researchers have adopted the theory of planned behavior (TPB) proposed by Fishbein and Ajzen (1975). The theory is quite versatile. It has been applied to environmental protection–related research, such as green hotels (e.g., Han and Kim, 2010). The theory posits that behavioral intention is contributed to by attitude, subjective norm, and perceived behavior control (Ajzen, 1991). Researchers (Vermeir and Verbeke, 2006) also suggest additional antecedents, such as price consideration, convenience, and quality, to enhance prediction. Another interesting point to consider is the “interest” construct. Renninger (2000) suggests that an individual’s personal interest is a strong determinant for her/his intrinsic motive. Yet, most studies only focus on the attitude–behavior paradigm. There is a recent study (Siegel et al., 2019) that proves the moderating effect of interest on the attitude–behavior relationship. However, there are limited studies on the formation of interest itself. The goal of this study is to bridge a gap by examining the factors that contribute to consumers’ sustainable food buying interest. The aim of this study is, therefore, to explore consumers’ sustainable food buying interests. The study is also interested in understanding what contributes to the interest in buying sustainable food. The study is focused on interest instead of intention or behavior to better understand the formation of interest.

## Literature Review

### Sustainable Food

Sustainable food is food produced by using sustainable agriculture, which is farming in sustainable ways including efficient land usage and environment-friendly farming techniques (Vermeir and Verbeke, 2006). Sustainability also addresses reducing food waste, lowering production cost, producing healthier food, and maintaining economic profit for the suppliers (Vermeir and Verbeke, 2006; Hobbs, 2007; McCarthy, 2014). Although this new wave of consumerism mainly focuses on environmental issues, it also incorporates other considerations such as animal welfare, human rights, and labor working conditions (Henderson, 2004; Vermeir and Verbeke, 2006). The goal is certainly admirable, but studies have shown conflicting results. For example, there are studies (e.g., Sobal, 2017; Hsu et al., 2018) that suggest that an individual’s food choice is rarely influenced by altruistic motives for the environment and/or livestock, while other studies have shown the exact opposite (e.g., Sellitto et al., 2018). This suggests that sustainable food consumption behavior is still an understudied issue. This is especially true in countries where environmental awareness has just begun to take root (Hoek et al., 2011). Daily food consumptions are still dictated by egoistic factors, such as convenience, habit, price, health benefits, enjoyment, and subjective norms (Vermeir and Verbeke, 2006). Meanwhile, the drive to push individuals to electively accept new dietary choices is hindered by factors such as benefit uncertainty (Kim and Iwashita, 2016; Fennell and Bowyer, 2019), rejecting unfamiliar products, and decreased taste satisfaction (Hoek et al., 2011).

A problem that may be brought about by the advancement is the mistreatment of livestock (Robbins, 2012) to improve the quality of food products. In order to maintain quality and increase production, genetic and pharmacologic manipulation is common (Tagliabue, 2017). In terms of environmental impact, Steinfeld et al. (2006) show that livestock account for 18% of the overall greenhouse gas emissions. There is also an assertion that the projection is an underestimate (Goodland and Anhang, 2009; Lee et al., 2014). Regardless, consuming less meat is one of the ways to slow down global warming. Furthermore, people must address the matters being raised. One is that genetically modified products tend to cause certain illnesses, such as neurodegenerative disorders (Holm et al., 2016). Another is the use of chemicals polluting land (Vermeir and Verbeke, 2006). And, there is livestock consuming copious amount of food (Nellemann, 2009; Fotiadis et al., 2019b), resulting in the need for more farmable land and subsequent ecological destruction.

In short, in order for humans to achieve a healthier diet and maintain ecological balance, a new concept regarding consuming sustainable food is identified as sustainable consumption. However, people still prefer meat products when they know for a fact that consuming more vegetables is healthier (Hoek et al., 2011). Despite the growing awareness of the need for environmental protection and the need for healthier living (Seyfang, 2006; Fotiadis et al., 2016a), the demand for sustainable food remains a niche market that is only able to attract customers with a certain profile (Fotiadis et al., 2016b).

### Level of Knowledge

In the studies relating to taking environmentally friendly actions, lack of knowledge is one of the common barriers. In the study of Chan (2008), hoteliers were reluctant to adopt an environmental management system because of the lack of knowledge and skill. The study of Han et al. (2009) concluded that hotel customers’ perception was affected by their knowledge about green practices, and that consequently dictated their behavior to some degree. Environmental knowledge can also change how customers perceive green marketing efforts of service suppliers (Yeh et al., 2016). Regarding knowledge, the level of wine-related knowledge was used as a segmentation tool to categorize customers to predict their wine purchasing behavior (Mitchell and Hall, 2004).

Knowledge matters in predicting individuals’ decision-making and subsequent behaviors. Knowledge is specifically pertinent to this study because the phenomenon we are examining is still in the process of change. For many countries, the environmental movement is just growing. The concept of sustainable food is intriguing but still foreign to many people. For them to accept a new concept and form new beliefs enough to change their present course of action is a challenge (Habermas, 2015). This is why an adequate level of knowledge is critical in promoting interest in sustainable food.

The study of environmental education states that environmental education can be categorized into five different phases, which include awareness, knowledge, attitude, skills, and participation (Stapp et al., 1969; Yeh et al., 2016). Given the rise of the environmental movement in many countries, it is safe to assume that we are at least at the “awareness” stage (Ballantyne et al., 2008; Chan et al., 2014). The linkage between knowledge and subsequent behavior is still weak (Roczen et al., 2014; Fotiadis et al., 2019a). This study examines the transition from knowledge to interest.

Currently, there are many misconceptions about sustainable food or even just the concept of sustainability. The misconceptions can hinder the effort to achieve sustainability (Muthu, 2017; Yin and Laing, 2017). Adequate knowledge helps individuals to make good decisions. This study assessed the level of knowledge that the respondents possessed regarding sustainable food in two ways. First, the respondents were asked to self-evaluate their own level of understanding in the matter. Second, respondents were asked a series of questions derived from a list of common misconceptions about sustainable food (Lemonick, 2009). This allowed the study to gauge the environmental knowledge of the respondents.

### Family and Friend Support

The opinion of someone important to an individual can greatly influence her/his views and decisions. There are many concepts describing the influence of group on an individual. For example, in the TPB model, subjective norm is used. Peer pressure and social pressure (Cui et al., 2016) are both terms used to describe individuals being influenced by their peers. Individuals often change their attitudes, values, and behaviors to conform to those of their peers. People are often seeking acceptance or approval from others (Hamilton et al., 2016); hence, other people’s opinion can affect choices of individuals.

Many researchers (e.g., Tarkiainen and Sundqvist, 2005; Han and Kim, 2010) posit that individuals’ behavioral intention is contributed to by three factors, namely, attitude, subjective norm, and perceived behavioral control. We use “attitude,” “subjective norm,” and “perceived behavioral control” in singular because they are being referred to as factors or constructs. In some studies, a linkage between subjective norm and attitude is proposed and tested. This raises the possibility of a mediating role of subjective norm between attitude and behavioral intention. In short, family and friend support can change the attitude and behavior of an individual. Some studies investigate the moderating effect of social opinion in the attitude–behavioral causal relationship (e.g., Al-Swidi et al., 2014). These studies suggest that attitude is, to a degree, shaped by social norm, and the relationships in the traditional TPB model are more complicated than usually modeled. If attitude is considered as an intrinsic motivation to perform or avoid an action, family and friend support is an extrinsic motivation (Mishkin et al., 2016).

### Price Accessibility

An individual’s choice can often be categorized into push and pull motives (Ryan et al., 2010). Individuals are pushed to certain choices by motivational factors but arrive at a choice by evaluating possible choices and selecting one that offers the best outcome or raises fewer objectionable concerns. This is where the construct of constraint comes in, or in TPB terms, perceived behavioral control. In many studies, price acts as either an incentive or a deterrent to a purchase decision (Tarkiainen and Sundqvist, 2005; Van Doorn and Verhoef, 2015; Paul et al., 2016). Han and Kim (2010) applied this in the study of green consumption and found price to be a strong determinant of behavioral intention. In other words, price can play a major role in decision-making.

### Health Incentive

One of the incentives for people to engage in sustainable consumption is health (Verbeke and Poquiviqui López, 2005). Especially in the current health-conscious world, more and more people prioritize personal health when making decisions (Potvin and Hasni, 2014; Manna and Jain, 2015; Radnitz et al., 2015). People are starting to desire leaner meat (Verbeke and Poquiviqui López, 2005), organic choices, and non–genetically modified products (Tarkiainen and Sundqvist, 2005). A healthy lifestyle is often linked with a sustainable environment; hence, the term LOHAS (lifestyles of health and sustainability) is introduced (Paul et al., 2016). Therefore, health incentive is thought to be an important contributor to an individual’s decision to engage in sustainable consumption.

### Interest

Interest refers to the pleasure that one associates with the idea of the existence of an object or taking an action (Habermas, 2015). Although the concept of interest is not new, many scholars see it as a part of attitude (Ajzen, 2002; Mishkin et al., 2016) or even the same thing. In their study, Potvin and Hasni (2014) treated interest, motivation, and attitude as similar constructs. However, some studies suggest otherwise. Renninger and Hidi (2015) asserted that interest triggers productive engagement and the potential for optimal motivation. If one is interested in an action, performing the action provides her/him with meaning, and thus, she/he has a favorable attitude toward it (Kong et al., 2018). There is also a recent study that uses interest as a moderator for the attitude–intention causal relationship (Siegel et al., 2019). Based on the works cited, the authors accept that interest is a different concept than attitude, but it can play an important role in shaping behavior.

With the recent rise of health concerns amongst people, interest in healthier food is gaining momentum (Radnitz et al., 2015). Very few studies examine this emerging phenomenon and try to understand the related formation of interest. However, Hung et al. (2016) postulated that individuals’ knowledge is an indicator of interest in new meat products. The main focus of their study was, however, still the traditional attitude–intention paradigm. Interest remained a small part of their investigation and a part of the attitude construct. Tarkiainen and Sundqvist (2005) studied organic food buying behavior in Finland. In their study, terms such as personal and collective interest were used. However, the authors only mentioned them in literature section and never incorporated the concept of interest in their research design. Verbeke and Poquiviqui López (2005) studied ethical food choice in Belgium. In this study, the concept of interest has been properly explored. The authors suggested that the determinants of interest include socio-demographics, food neophobia, acculturation level, and openness to new things. From these factors, one can see that the authors attributed the formation of interest to personality traits, such as socio-economic status and personal preference. The study also asserted the importance of social factors relating to personal interest, which can affect food choice. The fear of new things (i.e., neophobia) is another interesting issue in their study. Neophobia is a frequently researched topic in food choice studies and is known to have effect on attitude (Huang et al., 2019).

As previously stated, interest is the positive feeling one gets when performing certain behaviors (Habermas, 2015). It then stands to reason that before a positive attitude can be formed and motivation aroused, an individual must first have interest in a subject. This is especially true when an individual is exposed to a new and unfamiliar subject (Verbeke and Poquiviqui López, 2005; Huang et al., 2019) where a lot of uncertainty exists. Uncertainty can dampen desire (Vermeir and Verbeke, 2006). The point is that it is important to examine the factors that contribute to the increase of interest.

## Research Method

### Research Framework and Hypotheses

**FIGURE 1:**
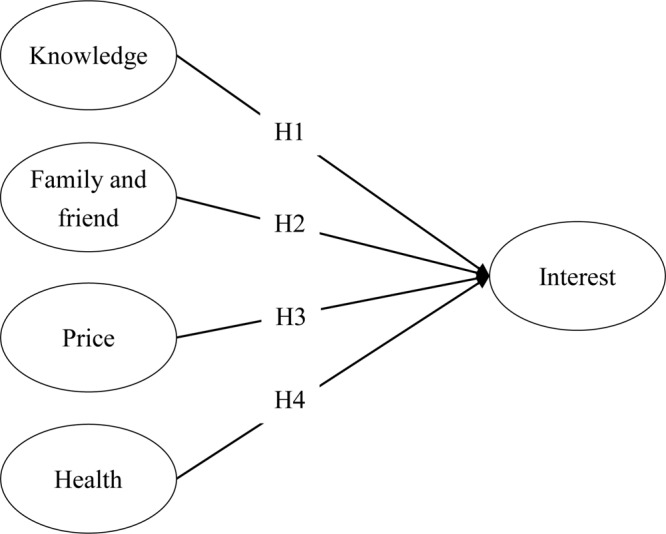
Research framework.

Past studies, such as that of Hung et al. (2016), indicate that individuals’ level of knowledge can influence their interest in a subject. A similar study shows that interest and knowledge show a high level of correlation (Hvenegaard, 2017). There are also other studies, such as those of Mitchell and Hall (2004) and Yeh et al. (2016), suggesting that knowledge is an important determinant of behavior. Even though most studies do not link knowledge directly to interest, the causal relationship between the two is an intriguing topic. It is clear that level of knowledge increases individuals’ interest in a certain subject. Therefore, the study proposes a research framework as shown in Figure 1 and hypothesizes that:

H1: Individuals’ knowledge positively increases their level of interest in buying sustainable food.

Research on TPB has established that subjective norm affects intention (Ajzen, 2002), and in some studies (e.g., Tarkiainen and Sundqvist, 2005; Han and Kim, 2010), subjective norm affects attitude too. Hassan et al. (2019) also suggest that social influence often interacts with personal interest and has the tendency to merge. This shows that individuals’ interests are often reshaped by collective interest. In fact, there are several terms specifically designated for the phenomenon, such as bandwagon effects (Liu et al., 2018), conformity (Smith and Haslam, 2017), and herd behavior (Kameda and Hastie, 2015). Therefore, the study hypothesizes that:

H2: Individuals’ friends and their support increase their level of interest.

In most of the studies regarding constraints (e.g., Tarkiainen and Sundqvist, 2005; Van Doorn and Verhoef, 2015), price is a common factor investigated. Aschemann-Witzel and Zielke (2017) indicated that price is a major barrier to purchasing organic food (see also Aschemann-Witzel and Zielke, 2017). However, that does not address the link between price concern and interest. Therefore, the study hypothesizes that:

H3: Individuals’ price acceptability increases their level of interest.

With some of the food-related problems, such as obesity (Hwang et al., 2006; Maiano et al., 2016) or genetically modified food (Holm et al., 2016), health concern is a major incentive (Vermeir and Verbeke, 2006; Radnitz et al., 2015) or (Tagliabue, 2017) deterrent for food choice. Seo et al. (2016) pointed out that health-related factors can influence people’s willingness to buy. Therefore, the study hypothesizes that:

H4: Individuals’ health incentive increases their level of interest.

### Sampling Method

Due to time and resource constraints, the study utilized an internet survey platform named “DoSurvey” to distribute the questionnaire. Given that this paper aims to understand the behavior of Taiwanese citizens, the survey request was sent to Taiwanese forums, social media, and websites. The survey was conducted in 2016 from March to April. Survey invitations were sent out in a snowball manner. Invitations were posted in multiple online communities and forwarded by various members to other sites. In total, 333 usable questionnaires were obtained. Given that the study proposes a model containing five constructs, more than 300 responses is considered adequate for the analysis (Yeh et al., 2016). Because the study utilized an online survey, there is no need to consider the response rate.

### Questionnaire Design

The questionnaire has six sections. One of the sections is designed to collect basic information about the respondents. The other sections are designed to collect information about respondents’ level of knowledge, family and friend support, price acceptability, health incentive, and interest.

Constructs have the following basis in the literature. The level of knowledge construct is composed of a series of yes/no questions and another two items, based on Han et al. (2009), Lemonick (2009), Hung et al. (2016), and Yeh et al. (2016). The family and friend support construct is composed of seven items, based on Han and Kim (2010), Godbey et al. (2010), and Cui et al. (2016). The price acceptability construct is composed of two items, based on Godbey et al. (2010) and Paul et al. (2016). The health incentive construct is composed of three items, based on Verbeke and Poquiviqui López (2005), Hung et al. (2016), and Paul et al. (2016). The interest construct is composed of four items, based on Renninger and Hidi (2015) and Verbeke and Poquiviqui López (2005). Most of the questions in these sections have five-point Likert scale responses. Details of items and their descriptive statistical outputs are shown in Table 1.

**TABLE 1:** Items used in the survey.

**Construct/Items/References**	**Mean**	**SD**
Level of knowledge*; Cronbach’s α = 0.868; CR = 0.770; AVE = 0.528 Han et al. (2009), Lemonick (2009), Hung et al. (2016), and Yeh et al. (2016)		
I know a lot about sustainable food	2.77	0.92
I know where to purchase sustainable food	2.62	1.16
I know where to park my car when visiting	3.15	1.02
**Family and friend support**; Cronbach’s α = 0.828; CR = 0.875; AVE = 0.505 Godbey et al. (2010), Cui et al. (2016), Han and Kim (2010), and Paul et al. (2016)		
My friends support my decision to purchase sustainable food	3.21	1.03
My family supports my decision to purchase sustainable food	3.88	0.85
People I know support my decision to purchase sustainable food	3.39	0.96
People who purchase sustainable food are a community	3.25	0.92
I can share my interest of sustainable food with people I know	3.40	0.98
Public opinion for more use of sustainable food is growing	3.20	0.95
I know a lot of people who purchase sustainable food	2.41	1.08
**Price acceptability**; Cronbach’s α = 0.773; CR = 0.862; AVE = 0.757 Godbey et al. (2010) and Paul et al. (2016)		
I am willing to accept that sustainable food costs more	2.89	1.03
I am willing to re-budget if I want to purchase sustainable food	3.13	1.06
**Health incentive**; Cronbach’s α = 0.761; CR = 0.876; AVE = 0.711 Verbeke and Poquiviqui López (2005), Hung et al. (2016), and Paul et al. (2016)		
Sustainable food is healthier	3.72	0.90
Sustainable food is leaner	3.67	0.95
Sustainable food can improve my dietary balance	2.79	1.13
**Interest**; Cronbach’s α = 0.857; CR = 0.770; AVE = 0.528 Verbeke and Poquiviqui López (2005), Renninger and Hidi (2015)		
I often search for information about sustainable food	3.42	0.88
I like to talk to people about sustainable food	2.76	1.02
I am interested in sustainable food	3.60	0.96
I am interested in purchasing sustainable food	3.49	0.95

*Series of yes/no questions designed to assess respondents’ knowledge regarding sustainable food; see Table 3. CR, construct reliability; AVE, average variance extracted.

## Results

### Sample Characteristics

The gender distribution of the sample was relatively equal between male (53.2%) and female (46.8%). About half of the respondents (52%) were between 18 and 24 years old. Respondents between 45 and 54 years old accounted for 15.3% of the sample, and respondents above 55 years accounted for 14.7% of the sample. Respondents between 25–34 and 35–44 years old each accounted for about 9% of the sample. In terms of education level, 70.6% of the respondents possessed a university degree, and 12.9% had a postgraduate degree. This indicates that the respondents were highly educated. Of the respondents, 61.6% were single. As for monthly disposable income, 30.3% of the respondents had NT$5,001–10,000; 20.4% of the respondents had NT$10,001–30,000; 19.2% of the respondents had NT$3,001–5,000; and 15.3% of the respondents had NT$30,001 or more. US$1 is around NT$30 (New Taiwan Dollar).

### Reliability and Validity of Measurement

The questionnaire information on level of knowledge, family and friend support, price acceptability, health incentive, and interest has a five-point Likert scale response. The Cronbach’s α values of the constructs are all above the suggested acceptable level of 0.7 (Nunnally, 1978). Fornell and Larcker (1981) suggested that composite reliability should reach 0.6 and average variance extracted should reach 0.5. Table 1 shows that appropriate values were attained. The wording of items and CR (construct reliability) values are presented in Table 1. Discriminant validity information is also provided in Table 2. The values are adequate (Gefen and Straub, 2005).

**TABLE 2:** Discriminant validity.

	**FFS**	**HI**	**IT**	**LK**	**PA**
Family and friend support (FFS)	0.710				
Health incentive (HI)	0.533	0.843			
Interest (IT)	0.663	0.561	0.745		
Level of knowledge (LK)	0.439	0.221	0.368	0.727	
Price accessibility (PA)	0.562	0.304	0.365	0.517	0.87

One portion of questions designed to assess respondents’ knowledge level in sustainable food is a series of yes/no questions. The questions were developed considering some of the common misconceptions about sustainable food (Lemonick, 2009). These questions are listed in Table 3. From the table, one can see that most questions were widely known. Questions 2, 3, 4, 6, 8, and 11 were answered accurately by more than 80% of the respondents. There were, however, still about one-third to one-half of the respondents who possessed misconceptions regarding questions 1, 7, 9, 12, and 13. Questions 5 and 10 identify two of the most misunderstood matters.

**TABLE 3:** Questions to assess respondents’ level of knowledge.

**Questions**	**Accurate**	**%**
Sustainable food is vegan.	240	72.1%
Sustainable food helps to decrease carbon emission by reducing meat consumption.	302	90.7%
Sustainable food may be nutrition deficient.	299	89.8%
Sustainable food may be nutrition imbalanced.	285	85.6%
Sustainable food is more humane.	130	39.0%
Sustainable food is healthier.	307	92.2%
Sustainable food helps to prevent cancer.	186	55.9%
Sustainable food is good for the environment.	299	89.8%
Sustainable food is without meat product.	225	67.6%
Sustainable food helps to alleviate global warming.	142	42.6%
Sustainable food helps to alleviate famine in the world.	288	86.5%
Sustainable food requires less labor.	214	64.3%
Sustainable food costs more money.	162	48.6%
		

**FIGURE 2:**
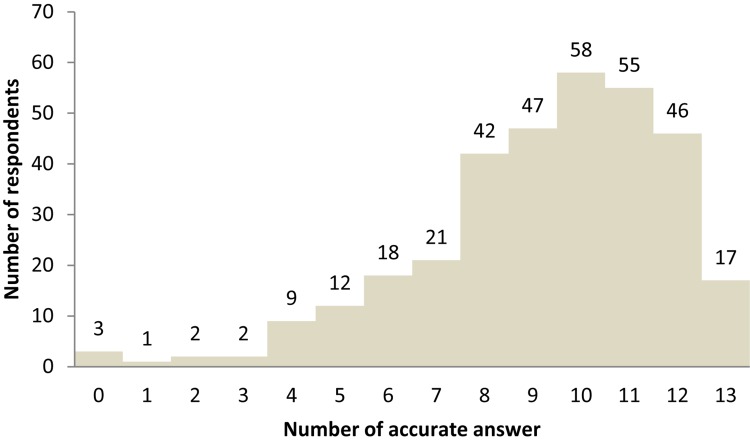
Respondents’ knowledge level.

From Figure 2, one can see that most respondents possess at least a moderate level of knowledge about sustainable food. Curiously, there were three respondents who answered all the questions wrong. Most respondents were able to get more than eight accurate responses, indicating a moderate to high level of knowledge. Therefore, the sample has respondents with a level of knowledge about sustainable food that skews high.

### Hypotheses Testing

The study utilized structural equation modeling (SEM) to examine the proposed causal relationships. The partial least squares (PLS) approach was used, which focuses on the analysis of variance (Wong, 2013). The study used a software named “SmartPLS” specifically designed to run PLS based on SEM (PLS-SEM) analysis.

The PLS-SEM uses different indices for model fit than conventional SEM. The conventional SEM is covariance-based, while PLS path modeling maximizes a correlation-based criterion (Henseler and Sarstedt, 2013). It is suggested that the standardized root mean square residuals (SRMRs) be lower than 0.08 and normed fit index (NFI) be higher than 0.8 (Henseler et al., 2016; Ramayah et al., 2017). The SRMR was equal to 0.053, and NFI was equal to 0.896. Both indices met the requirement given for the software.

**FIGURE 3:**
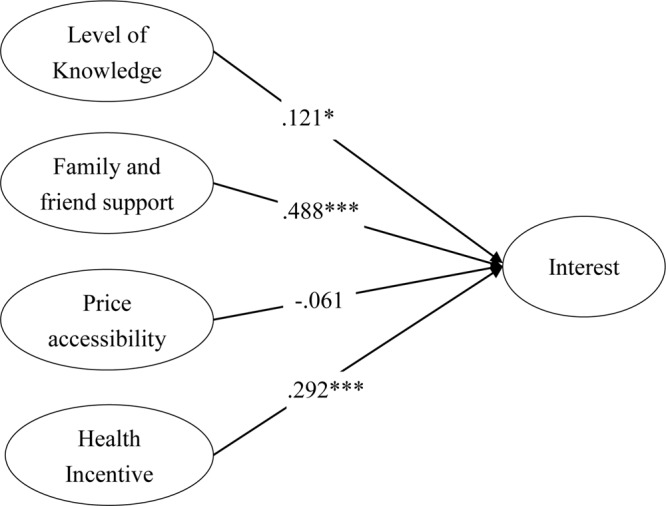
Result of analysis.

The result of estimation is presented in Figure 3. The result indicates that three of the four paths are significant. The first one hypothesizes the causal relationship between knowledge and interest. The β value is.121 with a probability of.013. This means that the level of knowledge significantly contributes to the level of interest at a moderate level. Therefore, hypothesis H1 is supported. The second path links family and friend support to level of interest. The β is.488 with a probability less than 0.001. It is the most significant contributor for interest in terms of β being significant and the value of the coefficient. Therefore, hypothesis H2 is supported. The third path is for the linkage between price acceptability and interest. The β value is equal to -0.061, but at an insignificant level. This means that hypothesis H3 is not supported. Finally, the linkage between health incentive and interest has a β value of.292 with a probability of 0.001. This finding supports H4.

## Discussion and Conclusion

### Theoretical Implications

The goal of this study is to understand factors contributing to people’s interest in sustainable food. The idea is to modify TPB (Ajzen, 1991), a model famous for studying human decision-making. In order to design a research tool specific to this study, some constructs were slightly modified. However, the idea remains similar. The subjective norm (Fishbein and Ajzen, 1975) was altered to family and friend support, and perceived behavior control (Ajzen, 2002) was replaced with price acceptability and health incentive. The idea is that the absence of obstacles piques the interest of respondents in sustainable food. The findings are, to an extent, similar to some from past studies. For example, knowledge is a powerful enough contributor for interest. While past studies often dealt with knowledge-to-intention (Chan, 2008; Han et al., 2009; Yeh et al., 2016) or knowledge-to-behavior (Mitchell and Hall, 2004) linkages, the result of this study indicates that knowledge also increases individuals’ interest. Similar findings have been derived in a tourism destination study (Lee and Bai, 2016), where respondents’ interest was generated from destination information.

### Managerial Implications

The construct of family and friend support, or subjective norm (Tarkiainen and Sundqvist, 2005; Han et al., 2009), showed a highly significant impact in terms of β on individuals’ level of interest. In fact, the construct was the strongest contributor to interest amongst the four predictors based on β means; this finding is consistent with those of some past studies (Tarkiainen and Sundqvist, 2005; Han and Kim, 2010). It is an indication that social norm is a key factor for new trends or movements to succeed. It is also one of the key concepts in TPB (Fishbein and Ajzen, 1975), where the opinion of important others can greatly affect one’s choice.

Price accessibility, however, does not appear to have significant impact on interest. This is different from most of the past studies (Tarkiainen and Sundqvist, 2005; Van Doorn and Verhoef, 2015), where price was often found to be a major factor of behavior. In fact, the finding of this study even yields an insignificant negative result. This suggests that behavior and interest are constrained in different ways. One way to look at this is that respondents often expected sustainable food to be pricier than conventional food. This can be seen in Table 3, where more than half of the respondents believed sustainable food costs more. If sustainable food costs less, this may lead to people dealing with questions or beliefs that are not being addressed. Many studies (e.g., Fritz et al., 2017) have found connections between authenticity and willingness to pay a price premium. Maybe it works in reverse, where authenticity is also judged by price tag.

Past studies (Tarkiainen and Sundqvist, 2005; Radnitz et al., 2015) often found healthiness of food to be one of the most frequently mentioned motives for food choice. The relationship between health consciousness and attitudes toward buying food has been established in some of the studies (Potvin and Hasni, 2014; Manna and Jain, 2015; Radnitz et al., 2015). The result of this study echoes those findings where health incentive is the second strongest contributor of the four. This suggests that individuals are often motivated by egoistic factors to guide their behavior and choice.

In summary, this study is meant to explore consumers’ sustainable food buying interest. The results of the present study show that family and friend support and health incentive are the two strongest predictors for sustainable food buying interest. Price accessibility, on the other hand, possesses no impact on consumers’ sustainable food buying interest. This is not to say that price accessibility does not influence consumers’ sustainable food buying interest. It just means that it is not the factor that fosters consumers’ sustainable food buying interest. Once individuals have sustainable food buying interest, they may still rely heavily on price evaluation in making sustainable food purchase choices.

### Limitations and Future Research

There are limitations in this research. First, the sample is likely composed of individuals with relatively high level of knowledge regarding sustainable food compared to a random sample from Taiwan’s population. It will be interesting to have results from individuals who are representative of Taiwan’s population or a particular segment there is a reason to study. In this study, an attempt at moderation analysis yielded no statistically significant results. A future study with a different sample should examine moderation again. The fact that the survey was conducted using an online survey platform is at the core of the limitations. A diverse, similar online survey that has more of a tendency to elicit respondents skeptical about sustainable food could allow examination of demographics to help understand where support and resistance come from. Finally, this study focuses on the determinants of the construct interest. Future studies could see how interest impacts attitude, intention, and behavior.

## Data Availability Statement

The raw data supporting the conclusions of this article will be made available by the authors, without undue reservation, to any qualified researcher.

## Ethics Statement

Ethical review and approval was not required for the study on human participants in accordance with the local legislation and institutional requirements. Written informed consent from the participants was not required to participate in this study in accordance with the national legislation and the institutional requirements.

## Author Contributions

S-YH, J-LH, and H-CC contributed to the development of research framework, conducted the survey, wrote, and searched for references. H-CW contributed to the research framework, designed the methodology and revised the manuscript.

## Conflict of Interest

The authors declare that the research was conducted in the absence of any commercial or financial relationships that could be construed as a potential conflict of interest.
